# Validity and reliability of the Physical Activity Questionnaire for Children (PAQ-C) and Adolescents (PAQ-A) in individuals with congenital heart disease

**DOI:** 10.1371/journal.pone.0175806

**Published:** 2017-04-26

**Authors:** Christine Voss, Paige H. Dean, Ross F. Gardner, Stephanie L. Duncombe, Kevin C. Harris

**Affiliations:** 1 Children’s Heart Centre, BC Children’s Hospital, Vancouver BC, Canada; 2 Department of Pediatrics, University of British Columbia, Vancouver BC, Canada; Vanderbilt University, UNITED STATES

## Abstract

**Objective:**

To assess the criterion validity, internal consistency, reliability and cut-point for the Physical Activity Questionnaire for Children (PAQ-C) and Adolescents (PAQ-A) in children and adolescents with congenital heart disease–a special population at high cardiovascular risk in whom physical activity has not been extensively evaluated.

**Methods:**

We included 84 participants (13.6±2.9 yrs, 50% female) with simple (37%), moderate (31%), or severe congenital heart disease (27%), as well as cardiac transplant recipients (6%), from BC Children’s Hospital, Canada. They completed the PAQ-C (≤11yrs, *n* = 28) or–A (≥12yrs, *n* = 56), and also wore a triaxial accelerometer (GT3X+ or GT9X) over the right hip for 7 days (*n* = 59 met valid wear time criteria).

**Results:**

Median daily moderate-to-vigorous physical activity was 46.9 minutes per day (IQR 31.6–61.8) and 25% met physical activity guidelines defined as ≥60 minutes of moderate-to-vigorous physical activity per day. Median PAQ-score was 2.6 (IQR 1.9–3.0). PAQ-Scores were significantly related to accelerometry-derived metrics of physical activity (*rho* = 0.44–0.55, all *p*<0.01) and sedentary behaviour (*rho* = -0.53, *p*<0.001). Internal consistency was high (α = 0.837), as was reliability (stability) of PAQ-Scores over a 4-months period (ICC = 0.73, 95%CI 0.55–0.84; *p*<0.001). We identified that a PAQ-Score cut-point of 2.87 discriminates between those meeting physical guidelines and those that do not in the combined PAQ-C and–A samples (area under the curve = 0.80 (95%CI 0.67–0.92).

**Conclusion:**

Validity and reliability of the PAQ in children and adolescents with CHD was comparable to or stronger than previous studies in healthy children. Therefore, the PAQ may be used to estimate general levels of physical activity in children and adolescents with CHD.

## Introduction

Congenital heart disease (CHD) affects approximately 1 in 100 live births [1]. Improvements in cardiac surgery and medical care have drastically improved survival and life expectancy for these children [2–4]. Consequently, the prevalence of CHD in children and adults across all age ranges has increased [5]. These individuals are at high cardiovascular risk as a direct result of their cardiac condition and the importance of physical activity is increasingly recognized and promoted to optimize long-term cardiovascular health and quality of life.

Physical activity is defined as ‘any bodily movement produced by skeletal muscles that results in energy expenditure’ [6]. The accurate assessment of physical activity is of paramount importance in order to: 1) better understand current physical activity levels in this at-risk patient population; 2) design strategies to maintain or improve physical activity; and 3) evaluate the effectiveness of such strategies. Valid and accurate objective measures of physical activity range from direct physiological assessments of energy expenditure (doubly-labeled water, direct room calorimetry), to assessments of bodily movement in free living conditions through wearable devices such as accelerometers and pedometers [7]. Self-report instruments (questionnaires, recall, diaries) provide a cost-effective and easy-to-use alternative to the expensive and burdensome objective measures, and can have acceptable validity depending on the self-report instrument [7]. Furthermore, structured questionnaires can provide useful insight into physical activity types and domains, which may be particularly relevant to assess behavioural changes over time and/or in response to physical activity interventions.

However, subjectivity, social desirability bias, and variable recall ability—especially in younger people–are considerable limitations of self-report instruments and establishing their validity against criterion measures is of critical importance. An expert panel recently ranked the Physical Activity Questionnaire for Children (PAQ-C) and Adolescents (PAQ-A) as one of very few self-report instruments that has acceptable validity, reliability and practicality for use in children and adolescents [8]. The PAQ is a self-administered, 7-day recall questionnaire designed to provide a general estimate of physical activity levels in healthy 8–20yr olds, derived from a series of questionnaire items on sport participation, activity during and after school, as well as in the evenings and at weekends [9]. Studies that have assessed the validity and reliability of the PAQ have been varied in study designs and populations, but have overall been convincing [10–18]; however, it is not known whether the PAQ is also valid for use in children and adolescents with CHD, given our limited knowledge regarding CHD children’s physical activity levels compared with healthy peers.

The objective of the current study was to assess the validity of the Physical Activity Questionnaire for Children (PAQ-C) and Adolescents (PAQ-A) in children and adolescents with CHD by assessing 1) its criterion validity against concurrently measured physical activity by accelerometry in children and adolescents with CHD; 2) its internal consistency and reliability over a 4-months period; 3) its ability to meaningfully discriminate by means of cut-point between those meeting physical activity guidelines vs those that do not (by accelerometry).

## Materials and methods

### Sample

We invited children and adolescents with CHD (including cardiac transplant recipients), aged 9–18yrs to participate in a physical activity intervention study. We offered participation to individuals who met our inclusion criteria and who were seen at the Children’s Heart Centre at BC Children’s Hospital in Vancouver, Canada, or at select pediatric cardiology partnership clinics across British Columbia and the Yukon, Canada, between September 2015 and October 2016. We registered excellent recruitment success (*n* = 93; 97%) and we used baseline data for the current analyses. Prior to study commencement, we obtained written informed parent/guardian consent and written informed participant assent. The Children's and Women's Research Ethics Board at the University of British Columbia approved the study (H14-01184).

### Participant characteristics

We obtained participant sex, birth date and cardiac diagnosis from patient charts. Trained nurses or research assistants measured stature (0.1 cm) and body mass (0.1 kg). Body Mass Index (BMI; kg∙m^-2^) was calculated and expressed as age-sex percentile scores based on World Health Organization growth charts [19]. BMI weight category was defined as ‘underweight’, ‘normal weight’, ‘overweight’ or ‘obese’ according age-sex-specific cut-points by the International Obesity Task Force (<18 yrs), or as <18.5 kg∙m^-2^, 18-5-24.9 kg∙m^-2^, 25–29.9 kg∙m^-2^ and ≥30 kg∙m^-2^, respectively (≥18 yrs) [20,21]. CHD disease complexity was classified as mild (e.g. atrial septal defect, mild pulmonary stenosis), moderate (e.g. coarctation of the aorta, Tetralogy of Fallot), severe (e.g. Fontan circulation, transposition of the great arteries), or cardiac transplant according to consensus guidelines [22].

### Accelerometry: Objective assessment of physical activity levels

We fitted participants with an ActiGraph accelerometer (GT3X+, GT9X; ActiGraph LLC, Pensacola, FL)–a commonly used tri-axial accelerometer to objectively measure habitual physical activity levels in children. In order to use established methods to convert raw acceleration signals into meaningful physical activity intensities, and to allow for comparisons with existing national and international physical activity data on children and adolescents, we positioned the monitors over the right hip with an elastic belt (GT3X+) or clip (GT9X). We provided uniform instructions to continue wearing the monitor for the next seven consecutive days, and to only remove it for water-based activities (including showering) and during sleep. The day on which the accelerometer was fitted did not record any data (unbeknownst to participants) so that participants could become accustomed to wearing the device and to minimise any influence of reactivity (i.e. participants changing their behaviour because they are being monitored.

We used ActiLife v.6.13.2 (ActiGraph LLC, Pensacola, FL) for accelerometer initialisation (sampling set at 30Hz), and file download, processing and analysis. We generated 15s epoch.agd files from the raw.gt3x files, and used the wear time function in ActiLife to identify valid accelerometry files. We considered a day to be valid if it was worn for ≥600 min/d, permitting 60min of ≤ 2min of zeros to avoid discarding reasonable sedentary bouts as non-wear time. For the current analyses, we defined participants accelerometry data as valid if they met conservative inclusion criteria of ≥4 valid days, of which ≥3 weekdays and ≥1 weekend day(s) had to be valid; these criteria are thought to provide the most accurate and reliable estimate of children’s average physical activity levels [23,24].

For valid accelerometry files, we extracted mean daily values for the sum of axis 1 (vertical) acceleration counts (hereon in referred to as ‘counts’) as a measure of total physical activity, and counts per minute (hereon in referred to as ‘CPM’) as a measure of relative overall physical activity intensity. To convert acceleration counts into time spent in meaningful physical activity intensities, we applied Evenson cut-points [25], which are considered the most accurate at classifying physical activity intensity in this age group [26]. Specifically, we estimated mean daily minutes of moderate-to-vigorous physical activity (MVPA; ≥2296 CPM; includes vigorous) and vigorous physical activity (≥4012 CPM). We also estimated mean daily minutes spent sedentary (<100 CPM); because sedentary time is dependent on accelerometry wear time, we expressed sedentary time as a percentage relative to wear time (‘%Sed’ = daily sedentary time (min)/daily accelerometer wear time (min)) [27]. For the purposes of this analyses, we defined meeting or failing physical activity guidelines as ≥60 min or <60 min of MVPA/d, respectively, in line with daily recommendations in physical activity guidelines [28].

### Physical activity questionnaire

Participants completed the Physical Activity Questionnaire for Children (PAQ-C) or Adolescents (PAQ-A). The PAQ is a self-administered, 7-day recall questionnaire that assesses participation in different physical activities, as well as activity during PE, lunch break, recess (PAQ-C only), after school, in the evenings and at weekends [9]. The PAQ-C is recommended for individuals in grades 4–8, who still have recess as part of their usual school day routine and who could be between 8–14 yrs old depending on the assessment data during the school year; the PAQ-A is recommended for individuals in grades 9–12, who do not have recess and who could be between ages 14–20 yrs old depending on the assessment date during the school year [9]. Because this study was conducted in the Canadian province of British Columbia, where secondary school usually starts with grade 8 and does not routinely offer recess, and because this was not a school-based study with measurements scattered across the calendar year, we opted for a conservative age threshold of ≤11 yrs for the PAQ-C and ≥12 yrs for the PAQ-A. The questionnaire was self-administered in a quiet room during regularly scheduled routine clinic visits. Each of the 8 (PAQ-A) or 9 (PAQ-C) questionnaire items is scored between 1 (low) and 5 (high physical activity), and a mean score of all items constitutes the overall PAQ score. Questionnaire items 9 (PAQ-A) or 10 (PAQ-C) asks participants whether they were sick last week, or whether anything prevented them from doing normal physical activities. This item is not included in the calculation of the PAQ score, but rather aims to gauge whether the responses likely represent the individual’s usual physical activity levels. Re-test questionnaire data was available for a subset of participants who repeated the assessment at the end of the overall study, 4 months later.

### Statistical analysis

Descriptive statistics (frequencies (%), mean±SD, median (IQR)) were calculated for applicable variables. Between-sex differences were assessed by independent t-tests (normal continuous data), Mann-Whitney U Ranked Sum test (non-normal continuous data), or Pearson’s chi-squared test (categorical data). Associations between relevant continuous variables were assessed by Spearman’s rank correlation (*rho*) and visual inspection of scatter plots. To assess whether a PAQ-score cut-point can differentiate between individuals who meet physical activity guidelines, we generated a receiver operating curve (ROC), using PAQ-scores as the classifier and meeting physical activity guidelines as the true-status reference (≥60 min/d based on accelerometry); in case of a significant area under the curve, the coordinate with the greatest sum of sensitivity and specificity identifies the PAQ-Score cut-point with discriminatory value [29]. Internal consistency of the PAQ was assessed by Cronbach’s alpha. Between PAQ-Scores between time points were assessed by Wilcoxon signed-rank test, and test-retest reliability (stability over time) was assessed using intra-class correlations coefficients. All analyses were carried out using Stata v. 14.1 (Stata Corp LP, College Station, TX) and significance was set at *p*<0.05.

## Results

Eighty-four participants completed all in-person assessment and provided valid and complete PAQ responses (*n* = 28 completed the PAQ-C, and *n* = 56 completed the PAQ-A). Of those, *n* = 59 (70%) met conservative accelerometry inclusion criteria of ≥3 valid weekdays and ≥1 valid weekend day. Characteristics of the overall sample and stratified by availability of valid accelerometry data are shown in Table 1. Approximately 1 in 3 participants were overweight or obese, slightly higher than national data [30]. The median PAQ-score (PAQ-A and PAQ-C combined) was 2.6 (IQR 1.9–3.0) broadly in line what would be expected of boys and girls across this age range [31,32]. Median PAQ-C scores were higher than PAQ-A scores (3.0 (2.4–3.6) vs. 2.4 (1.7–2.8), *p* = 0.001), which is to be expected given the significant age differences between participants who were given the PAQ-C vs. the PAQ-A (10.5±1.0 vs. 15.1±2.2 yrs, respectively, *p<*0.001). Nineteen individuals (23%) reported that one or more days during the previous 7 days were not normal because of sickness (*n* = 14), injury (*n* = 2) or abnormal school routines (*n* = 1 suspended, *n* = 1 school break, *n* = 1 exams), but neither their PAQ-scores nor accelerometry-derived physical activity measures were significantly different from those that reported a normal week. The most commonly reported sports and activities (questionnaire item 1 of the PAQ) in order of popularity were: walking, running, playing tag, soccer, and dance. There were no significant differences in sample characteristics, including PAQ-scores, between those with valid accelerometry and those without (all *p*>0.05). There were no significant between-sex differences in sample characteristics for the entire sample, or those with valid accelerometry data (all *p*>0.05).

**Table 1:** Sample characteristics and physical activity levels, stratified by availability of valid accelerometry data.

	All (*n* = 84)	Valid accelerometry (*n* = 59)	No or insufficient accelerometry (*n* = 25)	*p*-value*
**Patient Characteristics**				
Age (yrs)	13.6±2.9	13.4±2.8	14.1±3.0	0.313
Male/Female	42/42	33/26	9/16	0.095
Stature (cm)	155.9±14.3	155.1±14.0	157.9±14.9	0.409
Body Mass (kg)	53.2±19.1	51.3±18.1	57.7±21.0	0.163
BMI Percentile^a^	61.8±32.6	59.3±32.6	68.1±32.2	0.259
BMI Category (Under-/Normal/Over-/Obese/missing)^b^	8/49/17/8/2	6/36/12/3/2	2/13/5/5/0	0.226
Cardiac Diagnosis (Mild/Mod/Severe/Tx)^c^	31/26/22/5	22/17/16/4	9/9/6/1	0.898
**Physical Activity Questionnaire (PAQ)**^d^				
PAQ-A/PAQ-C	56/28	36/23	20/5	0.092
PAQ Score	2.6 (1.9–3.0)	2.6 (2.1–3.1)	2.6 (1.6–3.0)	0.500
Walking ≥1/week (Y/N)^e^	71/13	48/11	23/2	0.217
Running ≥1/week (Y/N) ^e^	60/24	39/20	21/4	0.097
Playing Tag ≥1/week (Y/N) ^e^	47/37	35/24	12/13	0.339
Soccer ≥1/week (Y/N) ^e^	32/52	26/33	6/19	0.083
Dance ≥1/week (Y/N) ^e^	25/59	16/43	9/16	0.416
**Accelerometry-derived Physical Activity (PA)**^**f**^				
Valid wear days		6 (6–7)	-	
Steps (counts/d)		8,014 (6,478–10,623)	-	
Total PA (acceleration counts x10^3^/d)^g^		347 (266–437)	-	
PA Intensity (acceleration counts/min)		432 (329–535)	-	
Moderate-to-vigorous PA (min/d)^h^		46.9 (31.4–59.3)	-	
Vigorous PA (min/d) ^h^		13.6 (7.1–22.6)	-	
Sedentary Time (%/d)^i^		66.3 (60.3–74.4)	-	
Meets PA Guidelines (Y/N)^j^		14/45		

Date are mean±SD, *n*, or median (IQR); Tx–Cardiac transplant recipient

*between-group differences assessed by independent t-test, Pearson’s Chi-Squared test, or Mann Whitney U test

^*a*^ *B*ody *M*ass *I*ndex (kg∙m^− 2^); percentiles calculated based on age–sex specific WHO 2007 reference charts

^*b*^ *International Obesity Task Force* age–sex specific BMI weight categorization

^*c*^ Congenital heart disease severity classification in accordance with consensus guidelines

^d^PAQ—Physical Activity Questionnaire for Children (PAQ-C; ≤11yrs) and Adolescents (PAQ-A; ≥12 yrs), mean score of 8 (PAQ-A) or 9 (PAQ-C) questionnaire items scored on a scale from 1 (no/low activity) to 5 (very active)

^e^ Select activity participation frequently reported in the PAQ; frequencies recoded into Y (≥1/week) or N (never/week)

^f^ ActiGraph accelerometry (GT3X+ or GT9X; 60s epoch), data in this table are based on ≥ 4 days with ≥ 600 min valid wear time (incl. ≥ 1 weekend day)

^g^ Total activity—sum of axis1 (or vertical axis) counts/d

^h^ MVPA–mean daily min of moderate-to-vigorous physical activity (≥2,296 counts/min) and vigorous physical activity (≥4,012 counts/min)

^i^ Sedentary time (%)–mean sedentary minutes (≤100 counts/min) expressed as a percentage relative to valid monitor wear time

^j^ Less than (N) or greater than (Y) 60 minutes of MVPA/d

Accelerometry-derived physical activity metrics for those with valid accelerometry data (*n* = 59) are shown in Table 1. Physical activity levels tended to be broadly comparable to healthy Canadian children: group median MVPA was ~47 min/day and skewed group mean MVPA was no different from national data (~52 vs. ~50 min) [30]. Boys tended to have higher values for most accelerometry-derived physical activity metrics than girls, but this was significant only for total physical activity (*p* = 0.049) and mean daily vigorous physical activity (*p* = 0.008).

### Validity: Association between PAQ and accelerometry

**Fig 1:**
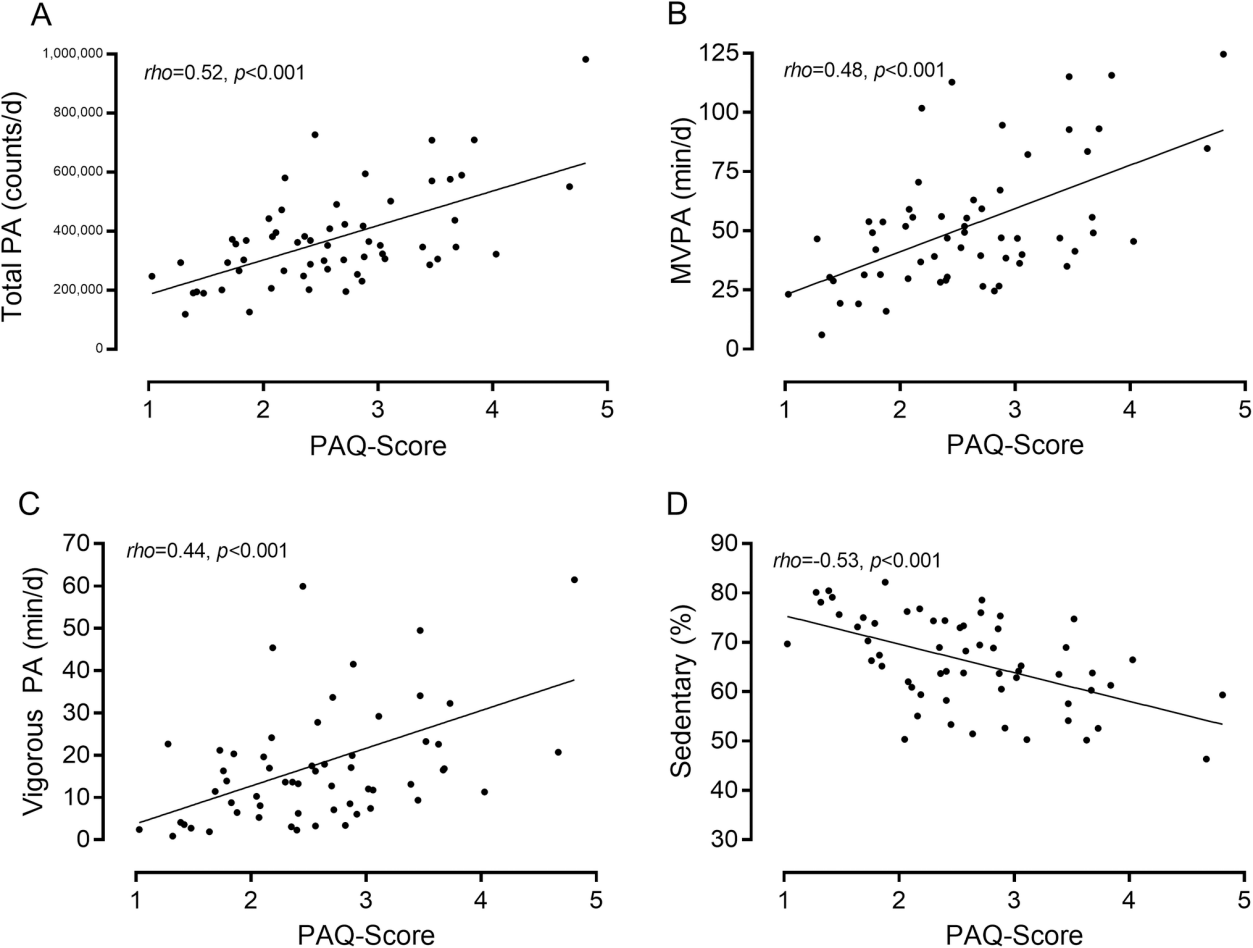
Association between PAQ-Score and various accelerometry-derived physical activity metrics.

Associations between PAQ-scores and accelerometry-derived physical activity metrics were computed for participants who met the most conservative accelerometry inclusion criteria (≥3 valid weekdays and ≥1 valid weekend day; *n* = 59). There were significant associations between PAQ-Scores, its sub-items and accelerometry-derived physical activity metrics (Table 2). Overall, associations were stronger for the overall PAQ-Score (–C or–A) than individual sub-items. Associations were overall similar if assessed within PAQ-C or PAQ-A respondents separately. Associations between the most pertinent accelerometry-derived physical activity metrics and overall PAQ-Scores are illustrated in Fig 1.

**Table 2:** Spearman rank correlations between accelerometry-derived physical activity metrics and PAQ-score items (*n* = 59).

	Total PA (counts/d)	PA Intensity (CPM)	MVPA (min/d)	Vigorous PA (min/d)	Sedentary Time (%/d)
PAQ-Score (–C or–A)	.51***	.54***	.47***	.44***	-.52***
Q1 –Checklist	.38**	.46***	.40**	.25	-.41**
Q2 –P.E.	.26*	.24	.20	.19	-.29*
Q3 –Recess^†^	.16	.32	.23	.17	-.21
Q4 –Lunch	.31*	.35**	.27*	.19	-.46***
Q5 –After School	.36**	.39**	.32*	.36**	-.32*
Q6 –Evenings	.38**	.40**	.43***	.37**	-.33*
Q7 –Weekends	.34**	.37**	.35**	.27*	-.38**
Q8 –Statement	.48***	.47***	.47***	.51***	-.35**
Q9 –Weekly	.52***	.50***	.50***	.45***	-.48***

* p<0.05

**p<0.01

***p<0.001

^†^ only *n* = 23 individuals completed the PAQ-C with the recess question (≤11 yrs) and had valid accelerometry data; interpret recess correlation coefficients with caution

PAQ–physical activity questionnaire; PA–physical activity; MVPA–moderate-to-vigorous physical activity

**Fig 2:**
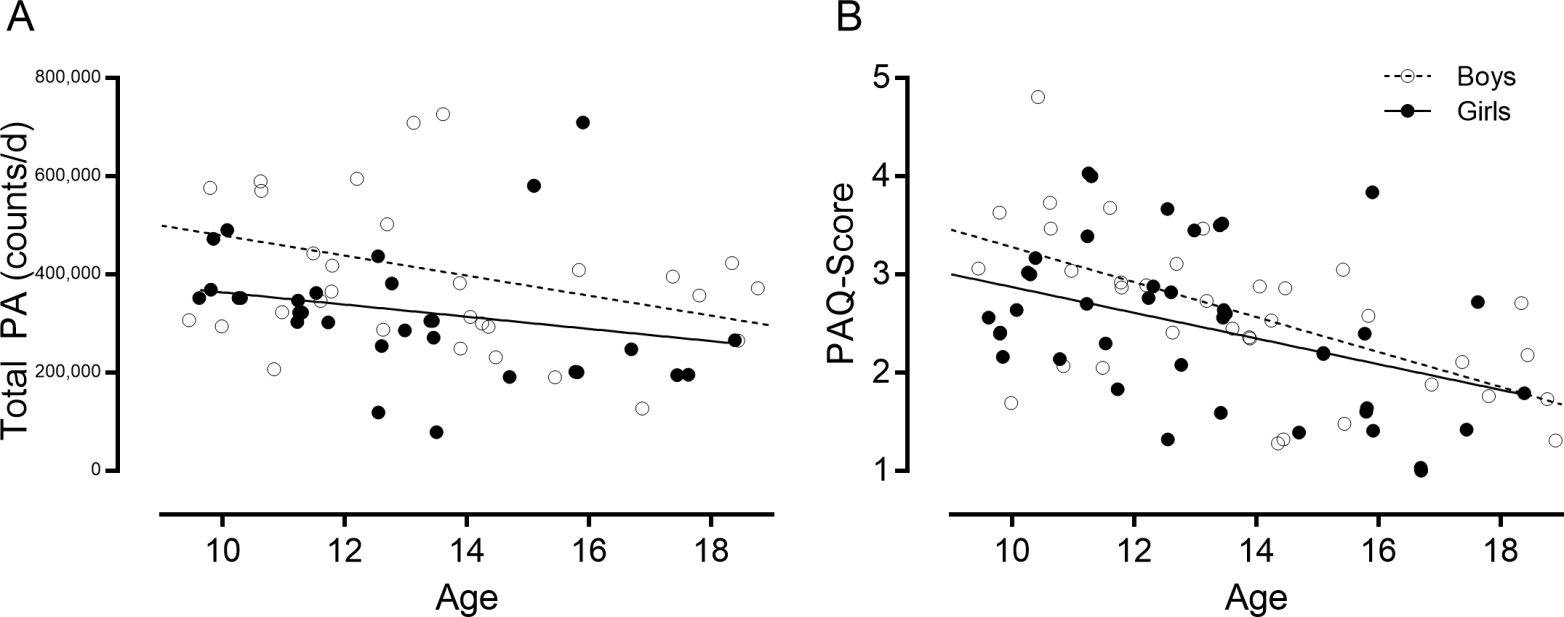
Association between age and physical activity captured by accelerometry and PAQ-score.

Fig 2 illustrates the inverse association between age and physical activity in both sexes. Older children and adolescents were significantly less active than younger children when measured by accelerometry (Panel A, total counts/d; boys *rho* = -0.32, girls *rho* = -0.51, both *p*<0.05) and according to the PAQ-score (Panel B, boys *rho* = -0. 63, girls *rho* = -0.45, both *p*<0.01).

### PAQ cut-points: Meeting physical activity guidelines

**Fig 3:**
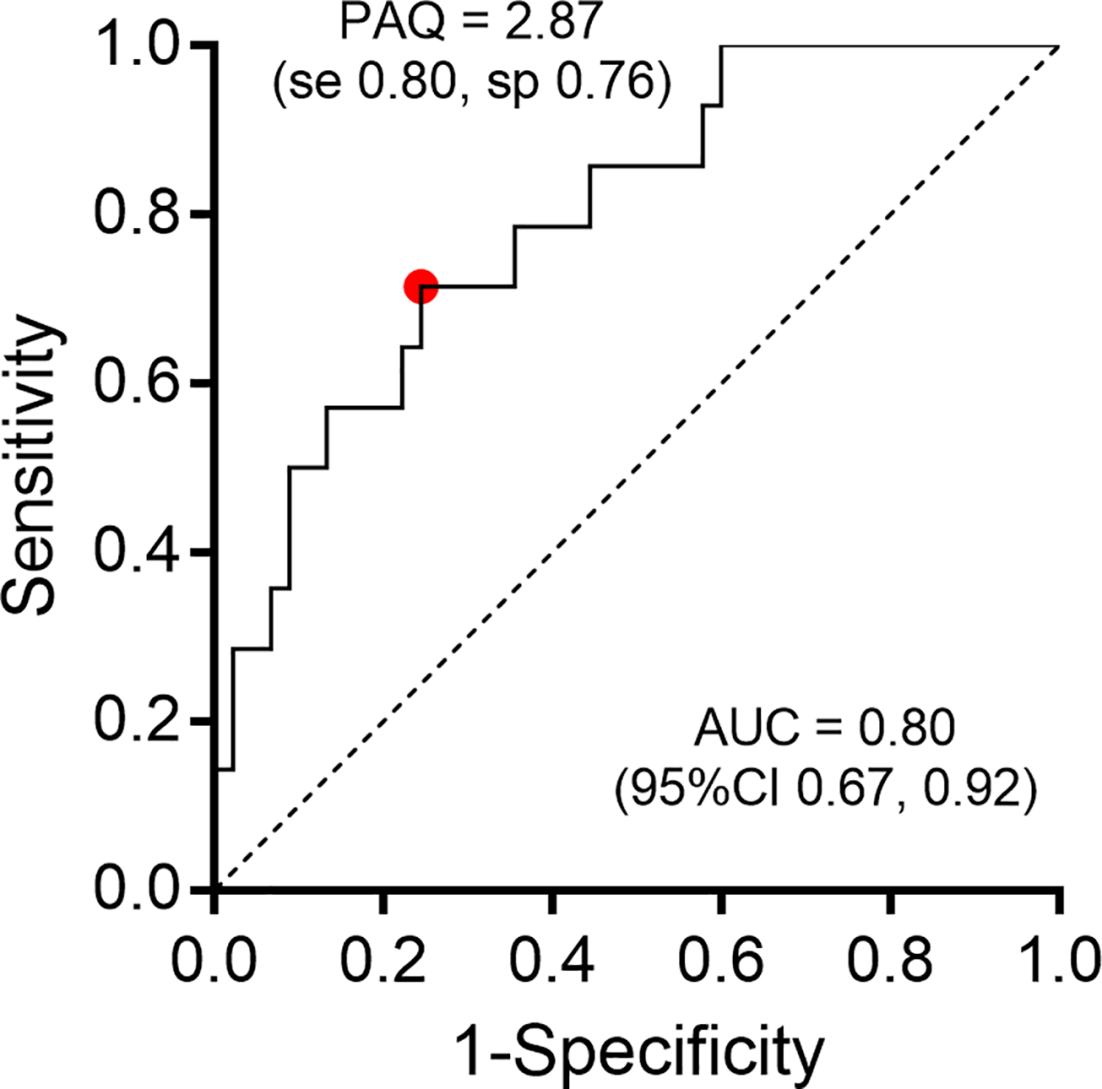
Receiver operating curve to identify PAQ-score cut-point that descriminates between those who meet physical activity guidelines (defined as ≥60 min MVPA/d based on accelerometry) versus those that do not.

A ROC curve was created using PAQ-scores as the classifier and meeting physical activity guidelines (≥60 min/d based on accelerometry) as the true-status reference (Fig 3). The ROC area under the curve (AUC) for the combined PAQ-C and–A samples was 0.80 (95%CI 0.67–0.92), and the coordinate with the greatest sum of sensitivity and specificity was 2.87, suggesting that this value may be used to crudely discriminate between individuals who are likely meeting physical activity guidelines and those who do not. Given the age-sex patterns in physical activity levels in our sample by both accelerometry and PAQ-score (Fig 2), it is likely that age-sex specific cut-points are warranted. Indeed, we found that the PAQ-C cut-point that corresponds to meeting physical activity guidelines in younger children was notably higher at 3.47 (AUC 0.73; 95%CI 0.49–0.97), although it is of note that this result was borderline insignificant, likely due to the relatively small sample size that completed the PAQ-C in our study (*n* = 23). For the PAQ-A (*n* = 36), the cut-point was 2.89 (AUC 0.81; 95%CI 0.63–0.99)–very comparable to the cut-point identified for the overall sample. We had insufficient cases to generate ROC curves by sex, and/or more nuanced stratification by age groups (e.g. older teens vs. younger teens, all of whom completed the PAQ-A).

### Reliability: Internal consistency and stability over time

Internal consistency of the questionnaire was high for PAQ-Scores overall (PAQ-A or–C combined, *n* = 84; α = 0.837), or within the PAQ-A (*n* = 56; α = 0.814) or PAQ-C responses separately (*n* = 28, α = 0.848). In the overall sample, corrected item-total correlations were high and ranged from *r* = 0.603 (Q5 after school) to *r* = 0.715 (Q9 weekly), with only the lunch (Q4) and PE question (Q2) being notably lower (*r* = 0.356 and *r* = 0.482, respectively). These patterns were similar when assessing the PAQ-C (ranging from *r* = 0.343 Q2 PE to *r* = 0.799 Q9 weekly) and PAQ-A (ranging from *r* = 0.230 Q4 lunch to *r* = 0.710 Q7 weekend). Removing the lunch and PE question marginally improved the internal consistency for the overall sample (α = 0.837 to 0.859), or when considering the PAQ-C (α = 0.848 to 0.863) or PAQ-A separately (α = 0.814 to 0.839). However, given that the internal consistency was high in the original version of the questionnaire (α>0.8) and improved only minimally by removing questionnaire items, as well as the fact that the school-based questions (lunch and PE) may provide useful insight into a different domain of a child’s physical activity behaviour and routine, we did not modify the original questionnaire.

PAQ-Score data at the 4-months follow up time point were available for a subset of participants (*n* = 45). PAQ-Scores were significantly different between baseline and follow-up (median 2.7 vs. 2.3, *p* = 0.041). Reliability (stability) of the overall PAQ-Scores over the 4-months was strong (ICC = 0.73, 95%CI 0.55–0.84; *p*<0.001).

## Discussion

In children and adolescents with CHD, we assessed the validity of the Physical Activity Questionnaire (PAQ-C and -A). Given our limited knowledge regarding CHD children’s physical activity levels compared with healthy peers, it was unclear whether the PAQ (a valid, reliable instrument in healthy children) is valid in this population. We demonstrated moderate-to-high criterion validity for the PAQ compared with physical activity measured concurrently by accelerometry, good internal consistency, and reliability over a 4-months period. Our data suggests that the PAQ is valid and useful to assess general levels of physical activity in children and adolescents with CHD.

### Validity: The PAQ demonstrates moderate-to-high criterion validity in CHD

In our sample of children and adolescents with CHD, we demonstrate moderate-to-high criterion validity for the PAQ compared with physical activity concurrently measured via accelerometry (acceleration counts/d: *rho* = 0.52; MVPA min/d: *rho* = 0.48). These findings are encouraging: our coefficients comparing the PAQ with accelerometry are notably higher than those in original validation studies in healthy Canadian children (*r* = 0.33 and *r* = 0.39) [12,13]. Our validity results are comparable or superior compared with other validation studies in international samples, such as validation studies in UK versions of the PAQ (total physical activity: *r* = 0.42, MVPA: *r* = 0.39) [10], in a Chinese sample (MVPA r = 0.33) [18], two Spanish samples (total physical activity: *rho* = 0.39, MVPA: *rho* = 0.34) [14] and (MVPA rho = 0.23) [33], and an Italian sample (MVPA *rho* = 0.30) [17]. The latter Italian study included a sub-study of reliability and construct validity in children with CHD (with favourable results), but the CHD children were not included in the assessment of criterion validity [17]. The reason associations were somewhat lower compared with our study could be because international validation studies either used the original Canadian version, or made modifications to make the questionnaire more suitable for the current cultural context (for example the specific sports on the activity checklist). It is unclear to what extent–if any—questionnaire modifications, or application of the original Canadian version in culturally diverse contexts impact associations with objectively measured physical activity levels. The suitability of the original physical activity checklist for our sample is evidenced by the fact that each activity was checked at least once by an individual in our sample, and that specification of ‘other’ activities were relatively rare, with most common responses being trampoline (n = 5), gymnastics (n = 4) or some variety of martial arts (n = 4).

Another criterion validation study in US children found coefficients of similar strengths to those in our study (total physical activity: *rho* = 0.56; MVPA: *rho =* 0.63), the reasons for which were partially attributed to refinements of the PAQ by the authors [11]. We used the original version of the PAQ [9] in our study and suggest several factors contribute to our convincing criterion validity. First, our sample of children and adolescents with CHD were able to complete the PAQ in a quiet setting and without time pressures. This is in stark contrast to the potential distractions and peer-pressures in the classroom setting for which the PAQ was designed and in which it was originally validated; our quiet setting may have contributed to more thoughtful and truthful answers in some children. Second, we used state-of-the-art equipment and rigorous data analyses protocols for the objective assessment of physical activity by accelerometry, thereby optimising the probability of capturing ‘true’ physical activity levels in our sample. Finally, the children and adolescents in our sample resemble a 'captive audience' that is likely highly committed to optimizing health outcomes for themselves and other individuals living with similar heart conditions, which may explain our high overall consent rate (97%), as well as excellent PAQ completion rates and good adherence to the accelerometry wear protocol (non-wear or insufficient wear of equipment is common in these types of studies). We further underscore the content validity of the PAQ by demonstrating for the first time significant correlation coefficients across the entire physical activity spectrum, including vigorous physical activity (*rho* = 0.44) and sedentary behaviour (*rho* = -0.52).

It is of note that correlation coefficients that describe the association between self-report and objective measures of physical activity in children and adolescents rarely exceed moderate strength. Social desirability bias, where participants respond in a way that is more favourably received by others, is always a concern for a self-report instruments, as are memory errors; in children, however, a predominant concern is that they developing cognitive abilities may limit their ability to think abstractly and perform detailed recall, in particular when asked to report time [34]. In light of this context, the moderate coefficients are interpreted to resemble *high* validity for the PAQ [8]. Thus, we are confident in the validity of the PAQ to estimate general levels of physical activity in children and adolescents with CHD.

### Reliability: Internal consistency and stability over time

We observed high internal consistency of the PAQ (α = 0.84) in our sample of CHD children, indicating good internal consistency. Our findings are comparable to the original reliability study (α = 0.83) [35] and compares to or exceeds values reported in most other studies [10,11,16,17,36]. Only one study of a modified version in Spanish reported inadequate internal consistency (α = 0.65) [14]. A sub-sample of children and adolescents in our study completed the PAQ again after 4 months and remarkably, we found good stability over this unusually long time-frame for test-retest reliability (ICC = 0.73). It is of note that the majority of studies that have assessed reliability coefficients do so over a one week period, for example the original reliability study (ICC = 0.75–0.82) [35], and other studies (ICC = 0.71 [14], ICC = 0.78 [10], ICC = 0.82 [18]). One study assessed the reliability between the PAQ-C and PAQ-A version over a 2-year period, and reported weaker but significant associations over time (*rho* = 0.30–0.39) [11]. Another study assessed the reliability on the same day a few hours apart, and expectedly found very high reliability coefficients (0.96) [33]. It is important to interpret the strength of a reliability coefficient in relation to the time interval over which the instrument was administered: the very strong coefficients over very short time intervals are desired and encouraging as they support the notion that the wording and design of the instrument elicits consistent responses from children in relation to their recall ability, while a longer time period is indicative of the fact that the instrument is able to capture a relatively stable ‘ranking’ of individuals’ physical activity over time. Our results suggest that the PAQ can provide such stable ranking estimates in our patients with CHD, especially in light of the fact that we would expect absolute questionnaire responses to differ over the course our long 4-months’ time period due to seasonal and other lifestyle changes.

### A PAQ cut-point to identify meeting physical activity guidelines

We found that the PAQ has moderate strength (area under the ROC curve 0.80) to discriminate between children and adolescents who are meeting physical activity guidelines of ≥60 minutes of MVPA per day versus those that do not, using accelerometry as the true-status reference. These results are similar to another study using similar methodologies to identify PAQ-Score cut-points that correspond to 60 minutes of objectively measured MVPA in Spanish children and adolescents, who identified a cut-point of 2.75 for children and 2.73 for adolescents. [37]. Our higher cut-point of 2.87 is remarkably similar to those identified in a large study of UK schoolchildren, which found that PAQ-Scores of ≥2.9 in boys and ≥2.7 in girls were linked to adequate aerobic capacity (as measured by a shuttle run test) [31]. In line with what others have reported [31,32], we noted important age-sex patterns in PAQ-Scores (Fig 2), possibly necessitating age-sex specific PAQ-Score cut-points for children and adolescents with CHD. We did not have sufficient children and adolescents to rigorously evaluate whether PAQ-Score cut-points for meeting physical activity guidelines vary by sex or more specific age groups (i.e. younger teens vs. older teens). We did, however, identify that the cut-point specifically based on the PAQ-A version was very similar to the cut-point derived for the combined PAQ-A and–C samples (2.89 vs. 2.87); participants who completed the PAQ-C appeared to have a higher cut-point of 3.47, although this result was borderline insignificant due to the relatively low sample size and needs to be interpreted with caution. Nevertheless, it is possible that the cut-point of 2.87 that we propose based on the combined PAQ-C and–A samples may be too conservative for younger children, who should be encouraged to strive for higher scores.

### Utility of the PAQ in children and adolescents with CHD in clinical practice and research

Children and adolescents with CHD are at high cardiovascular risk as a direct result of their cardiac condition, which in turn may additionally exacerbate traditional cardiovascular risk factors, such as low physical activity and obesity [38]. Physical activity has not been extensively evaluated in this special population. The established validity of the PAQ for children and adolescents with CHD will be immediately useful to both clinical practice and research. The PAQ is cost-effective and a practical instrument that is designed to be completed independently by participants in <20 minutes [9]. We have experienced excellent acceptance and completion rates for the PAQ in our CHD patients, who routinely complete the questionnaire in <10 minutes.

The PAQ-Score as a metric of physical activity, however, may seem somewhat unpractical when physical activity guidelines are based on achieving ≥60 minutes of MVPA per day. As previously discussed, the developing cognitive abilities of children limits their ability to accurately recall amount and duration of activities; the fact that the PAQ does not attempt to elicit such detailed information is likely instrumental to its valid estimates of *general* physical activity levels, while still proving useful to discriminate between active and inactive children and adolescents [34]. To address the PAQ’s inability to provide specific estimates of time spent in meaningful physical activity intensities, an equation study has been developed to convert PAQ-Scores to percentage of time spent in MVPA, factoring in both participant sex and age [15]. Whether the %MVPA equivalent is more useful than the PAQ-Score itself warrants further evaluation.

Normative PAQ data can aide the interpretation of PAQ-Scores, and provide context between individuals and within individuals over time. We know of only one study that used the PAQ in children with CHD in Italy, which reported significant differences in PAQ-Scores by CHD disease complexity [17]. We did not attempt to assess differences by disease complexity given the obvious age-sex differences. Larger datasets would be needed to provide age-sex-specific PAQ-Scores by CHD disease complexity. However, we demonstrated that the PAQ identifies age-sex patters of physical activity in children and adolescents with CHD similarly to patterns in PAQ-Scores seen in healthy children and adolescents [31,32], and CHD-specific norms may not be necessary.

The PAQ offers the additional advantage of being able to capture participation in specific types of activities (item 1, activity check-list) [8]. While our widely-accepted criterion measure (accelerometry) is able to measure duration, intensity and frequency of physical activity, it cannot (yet) identify what type of activity was performed. In our sample, participants reported that the most commonly performed activity expectedly was walking, and sex-specific patterns relating to soccer and dance participation were starting to emerge. This contextual detail is likely useful for physical activity counselling and exercise prescription, as well as for evaluations of physical activity interventions. The PAQ also provides some information on school-based physical activity including participation and effort during physical education. In line with previous research in healthy children [11], we found that the physical education item was not significantly related to overall physical activity in our study. Nevertheless, information on physical education participation and effort is likely still of interest in this population, given that some children with CHD perceive their inferior physical abilities compared with peers as limiting and may even have experienced bullying as a result.

### Strengths and limitations

This is the first study that has sought to establish the validity of the PAQ for children and adolescents with CHD. We are the sole care provider for children and adolescents with CHD across the province of British Columbia with a population of more than 4 million people. In combination with our excellent recruitment rate of 97%, our study population includes a broad range of children and adolescents with many forms of CHD. In addition, we employed rigorous protocols to process and analyse our criterion physical activity measure. Specifically, we included total physical activity defined as acceleration counts/d in our analyses and found similar results to minutes of MVPA. The acceleration counts as an accelerometry metric is not affected by data processing decisions, such as the epoch length (longer epochs ‘dilute’ the acceleration signal of younger children, who have more sporadic, short and high intensity bursts of physical activity).

However, there are several limitations in our study. Not all participants adhered to the accelerometry wear protocol, and although we found no evidence for systematic bias based on participant characteristics and PAQ-Scores, it is possible that the more active children and adolescents were more likely to wear the accelerometer. Further, in light of obvious sex-age differences, we were inadequately powered to explore whether PAQ-Scores differed by CHD disease complexity, and to determine detailed PAQ-Score cut-points that correspond to meeting physical activity guidelines according to sex and age. Finally, our reliability analyses spanned a 4-months period, and differences in questionnaire responses are not only likely, but to be expected as a result of for example, seasonal variations (baseline assessments were predominantly in fall, follow-up in winter). Thus, our reliability results may have limited usefulness. Nevertheless, the relative stability of PAQ-Scores over the 4-month period is encouraging.

## Conclusion

In children and adolescents with CHD, the PAQ may provide valid estimates of general levels of physical activity. The self-administered questionnaire is a cost-effective and easy-to-use alternative to burdensome objective measures, and may serve as a useful instrument to assess general levels of physical activity in children and adolescents with CHD, and to assess change over time, for example in response to a physical activity intervention. This area of study is of increasing interest in this special population and as such, having easily administered, valid measurement tools is important.

## References

[1] van der Linde D, Konings EE, Slager MA, Witsenburg M, Helbing WA, Takkenberg JJ, et al Birth prevalence of congenital heart disease worldwide: a systematic review and meta-analysis. J Am Coll Cardiol. 2011;58:2241–2247. doi: 10.1016/j.jacc.2011.08.025 22078432 10.1016/j.jacc.2011.08.025

[2] Marelli AJ, Mackie AS, Ionescu-Ittu R, Rahme E, Pilote L. Congenital heart disease in the general population: changing prevalence and age distribution. Circulation. 2007;115:163–172. doi: 10.1161/CIRCULATIONAHA.106.627224 17210844 10.1161/CIRCULATIONAHA.106.627224

[3] Boneva RS, Botto LD, Moore CA, Yang Q, Correa A, Erickson JD. Mortality associated with congenital heart defects in the United States: trends and racial disparities, 1979–1997. Circulation. 2001;103:2376–2381. 11352887 10.1161/01.cir.103.19.2376

[4] Warnes CA, Liberthson R, Danielson GK, Dore A, Harris L, Hoffman JI, et al Task force 1: the changing profile of congenital heart disease in adult life. J Am Coll Cardiol. 2001;37:1170–1175. 11300418 10.1016/s0735-1097(01)01272-4

[5] Marelli AJ, Ionescu-Ittu R, Mackie AS, Guo L, Dendukuri N, Kaouache M. Lifetime prevalence of congenital heart disease in the general population from 2000 to 2010. Circulation. 2014;130:749–756. doi: 10.1161/CIRCULATIONAHA.113.008396 24944314 10.1161/CIRCULATIONAHA.113.008396

[6] Caspersen CJ, Powell KE, Christenson GM. Physical activity, exercise, and physical fitness: definitions and distinctions for health-related research. Public Health Rep. 1985;100:126–131. 3920711 PMC1424733

[7] Warren JM, Ekelund U, Besson H, Mezzani A, Geladas N, Vanhees L. Assessment of physical activity—a review of methodologies with reference to epidemiological research: a report of the exercise physiology section of the European Association of Cardiovascular Prevention and Rehabilitation. Eur J Cardiovasc Prev Rehabil. 2010;17:127–139. doi: 10.1097/HJR.0b013e32832ed875 20215971 10.1097/HJR.0b013e32832ed875

[8] Biddle SJ, Gorely T, Pearson N, Bull FC. An assessment of self-reported physical activity instruments in young people for population surveillance: Project ALPHA. Int J Behav Nutr Phys Act. 2011;8:1 doi: 10.1186/1479-5868-8-1 21194492 10.1186/1479-5868-8-1 PMC3019119

[9] Kowalski KC, Crocker RE, Donen RM. The Physical Activity Questionnaire for Older Children (PAC-C) and Adolescents (PAQ-A) Manual Saskatoon, Canada: University of Saskatchewan; 2004.

[10] Aggio D, Fairclough S, Knowles Z, Graves L. Validity and reliability of a modified english version of the physical activity questionnaire for adolescents. Arch Public Health. 2016;74:3 doi: 10.1186/s13690-016-0115-2 26807217 10.1186/s13690-016-0115-2 PMC4724149

[11] Janz KF, Lutuchy EM, Wenthe P, Levy SM. Measuring activity in children and adolescents using self-report: PAQ-C and PAQ-A. Med Sci Sports Exerc. 2008;40:767–772. doi: 10.1249/MSS.0b013e3181620ed1 18317366 10.1249/MSS.0b013e3181620ed1

[12] Kowalski KC, Crocker PRE, Faulkner RA. Validation of the Physical Activity Questionnaire for Older Children. Pediatr Exerc Sci. 1997;9:174–186.

[13] Kowalski KC, Crocker PRE, Kowalski NP. Convergent validity of the physical activity questionnaire for adolescents. Pediatric Exercise Science. 1997;9:342–352.

[14] Martinez-Gomez D, Martinez-de-Haro V, Pozo T, Welk GJ, Villagra A, Calle ME, et al [Reliability and validity of the PAQ-A questionnaire to assess physical activity in Spanish adolescents]. Rev Esp Salud Publica. 2009;83:427–439. 19701574 10.1590/s1135-57272009000300008

[15] Saint-Maurice PF, Welk GJ, Beyler NK, Bartee RT, Heelan KA. Calibration of self-report tools for physical activity research: the Physical Activity Questionnaire (PAQ). BMC Public Health. 2014;14:461 doi: 10.1186/1471-2458-14-461 24886625 10.1186/1471-2458-14-461 PMC4055223

[16] Thomas EL, Upton D. Psychometric properties of the physical activity questionnaire for older children (PAQ-C) in the UK. Psychology of Sport and Exercise. 2014;15:280–287.

[17] Gobbi E, Elliot C, Varnier M, Carraro A. Psychometric Properties of the Physical Activity Questionnaire for Older Children in Italy: Testing the Validity among a General and Clinical Pediatric Population. PLoS One. 2016;11:e0156354 doi: 10.1371/journal.pone.0156354 27228050 10.1371/journal.pone.0156354 PMC4881960

[18] Wang JJ, Baranowski T, Lau WP, Chen TA, Pitkethly AJ. Validation of the Physical Activity Questionnaire for Older Children (PAQ-C) among Chinese Children. Biomed Environ Sci. 2016;29:177–186. doi: 10.3967/bes2016.022 27109128 10.3967/bes2016.022

[19] de Onis M, Onyango AW, Borghi E, Siyam A, Nishida C, Siekmann J. Development of a WHO growth reference for school-aged children and adolescents. Bull World Health Organ. 2007;85:660–667. doi: 10.2471/BLT.07.043497 18026621 10.2471/BLT.07.043497 PMC2636412

[20] Cole TJ, Bellizzi MC, Flegal KM, Dietz WH. Establishing a standard definition for child overweight and obesity worldwide: international survey. BMJ. 2000;320:1240–1243. 10797032 10.1136/bmj.320.7244.1240 PMC27365

[21] Cole TJ, Flegal KM, Nicholls D, Jackson AA. Body mass index cut offs to define thinness in children and adolescents: international survey. BMJ. 2007;335:194–201. doi: 10.1136/bmj.39238.399444.55 17591624 10.1136/bmj.39238.399444.55 PMC1934447

[22] Warnes CA, Williams RG, Bashore TM, Child JS, Connolly HM, Dearani JA, et al ACC/AHA 2008 Guidelines for the Management of Adults with Congenital Heart Disease: a report of the American College of Cardiology/American Heart Association Task Force on Practice Guidelines (writing committee to develop guidelines on the management of adults with congenital heart disease). Circulation. 2008;118:e714–833. doi: 10.1161/CIRCULATIONAHA.108.190690 18997169 10.1161/CIRCULATIONAHA.108.190690

[23] Trost SG, Pate RR, Freedson PS, Sallis JF, Taylor WC. Using objective physical activity measures with youth: how many days of monitoring are needed? Med Sci Sports Exerc. 2000;32:426–431. 10694127 10.1097/00005768-200002000-00025

[24] Carson V, Janssen I. Volume, patterns, and types of sedentary behavior and cardio-metabolic health in children and adolescents: a cross-sectional study. BMC Public Health. 2011;11:274 doi: 10.1186/1471-2458-11-274 21542910 10.1186/1471-2458-11-274 PMC3112118

[25] Evenson KR, Catellier DJ, Gill K, Ondrak KS, McMurray RG. Calibration of two objective measures of physical activity for children. J Sports Sci. 2008;26:1557–1565. doi: 10.1080/02640410802334196 18949660 10.1080/02640410802334196

[26] Trost SG, Loprinzi PD, Moore R, Pfeiffer KA. Comparison of accelerometer cut points for predicting activity intensity in youth. Med Sci Sports Exerc. 2011;43:1360–1368. doi: 10.1249/MSS.0b013e318206476e 21131873 10.1249/MSS.0b013e318206476e

[27] Gabel L, McKay HA, Nettlefold L, Race D, Macdonald HM. Bone architecture and strength in the growing skeleton: the role of sedentary time. Med Sci Sports Exerc. 2015;47:363–372. doi: 10.1249/MSS.0000000000000418 24983338 10.1249/MSS.0000000000000418

[28] Canadian Society for Exercise Physiology. 24-Hour Movement Guidelines for Children and Youth. Ottawa, ON. 2016. Available: http://www.csep.ca/CMFiles/Guidelines/24hrGlines/Canadian24HourMovementGuidelines2016.pdf

[29] Akobeng AK. Understanding diagnostic tests 3: Receiver operating characteristic curves. Acta Paediatr. 2007;96:644–647. doi: 10.1111/j.1651-2227.2006.00178.x 17376185 10.1111/j.1651-2227.2006.00178.x

[30] Colley RC, Garriguet D, Janssen I, Craig CL, Clarke J, Tremblay MS. Physical activity of Canadian children and youth: accelerometer results from the 2007 to 2009 Canadian Health Measures Survey. Health Rep. 2011;22:15–23. 21510586

[31] Voss C, Ogunleye AA, Sandercock GR. Physical Activity Questionnaire for Children and Adolescents: English norms and cut-off points. Pediatr Int. 2013;55:498–507. doi: 10.1111/ped.12092 23461812 10.1111/ped.12092

[32] Thompson AM, Baxter-Jones AD, Mirwald RL, Bailey DA. Comparison of physical activity in male and female children: does maturation matter? Med Sci Sports Exerc. 2003;35:1684–1690. doi: 10.1249/01.MSS.0000089244.44914.1F 14523305 10.1249/01.MSS.0000089244.44914.1F

[33] Benitez-Porres J, Lopez-Fernandez I, Raya JF, Carnero SA, Alvero-Cruz JR, Carnero EA. Reliability and Validity of the PAQ-C Questionnaire to Assess Physical Activity in Children. J School Health. 2016;86:677–685. doi: 10.1111/josh.12418 27492937 10.1111/josh.12418

[34] Welk GJ, Corbin CB, Dale D. Measurement issues in the assessment of physical activity in children. Res Q Exerc Sport. 2000;71:S59–73. 10925827

[35] Crocker PR, Bailey DA, Faulkner RA, Kowalski KC, McGrath R. Measuring general levels of physical activity: preliminary evidence for the Physical Activity Questionnaire for Older Children. Med Sci Sports Exerc. 1997;29:1344–1349. 9346166 10.1097/00005768-199710000-00011

[36] Bervoets L, Van Noten C, Van Roosbroeck S, Hansen D, Van Hoorenbeeck K, Verheyen E, et al Reliability and Validity of the Dutch Physical Activity Questionnaires for Children (PAQ-C) and Adolescents (PAQ-A). Arch Public Health. 2014;72:47 doi: 10.1186/2049-3258-72-47 25671114 10.1186/2049-3258-72-47 PMC4323128

[37] Benitez-Porres J, Alvero-Cruz JR, Sardinha LB, Lopez-Fernandez I, Carnero EA. Cut-off values for classifying active children and adolescentes using the Physical Activity Questionnaire: PAQ-C and PAQ-ACut-off values for classifying active children and adolescents using the Physical Activity Questionnaire: PAQ-C and PAQ-A. Nutr Hosp. 2016;33:564 doi: 10.20960/nh.564 27759968 10.20960/nh.564

[38] Kavey RE, Allada V, Daniels SR, Hayman LL, McCrindle BW, Newburger JW, et al Cardiovascular risk reduction in high-risk pediatric patients: a scientific statement from the American Heart Association Expert Panel on Population and Prevention Science; the Councils on Cardiovascular Disease in the Young, Epidemiology and Prevention, Nutrition, Physical Activity and Metabolism, High Blood Pressure Research, Cardiovascular Nursing, and the Kidney in Heart Disease; and the Interdisciplinary Working Group on Quality of Care and Outcomes Research: endorsed by the American Academy of Pediatrics. Circulation. 2006;114:2710–2738. doi: 10.1161/CIRCULATIONAHA.106.179568 17130340 10.1161/CIRCULATIONAHA.106.179568

